# Supplementary material for: IRF2 is a master regulator of human keratinocyte stem cell fate
Source: Nat Commun. 2019 Oct 14;10:4676. doi: 10.1038/s41467-019-12559-x (PMC6791852; doi:10.1038/s41467-019-12559-x)
Supplement: Supplementary file 1 — Supplementary Information [file 41467_2019_12559_MOESM1_ESM.pdf]

Supplementary Information for

IRF2 is a master regulator of human keratinocyte stem cell fate

Mercado *et al*,

This PDF file includes:

Supplementary Figures 1-17

Supplementary Table 1

Supplementary Figures

Supplementary Figure 1

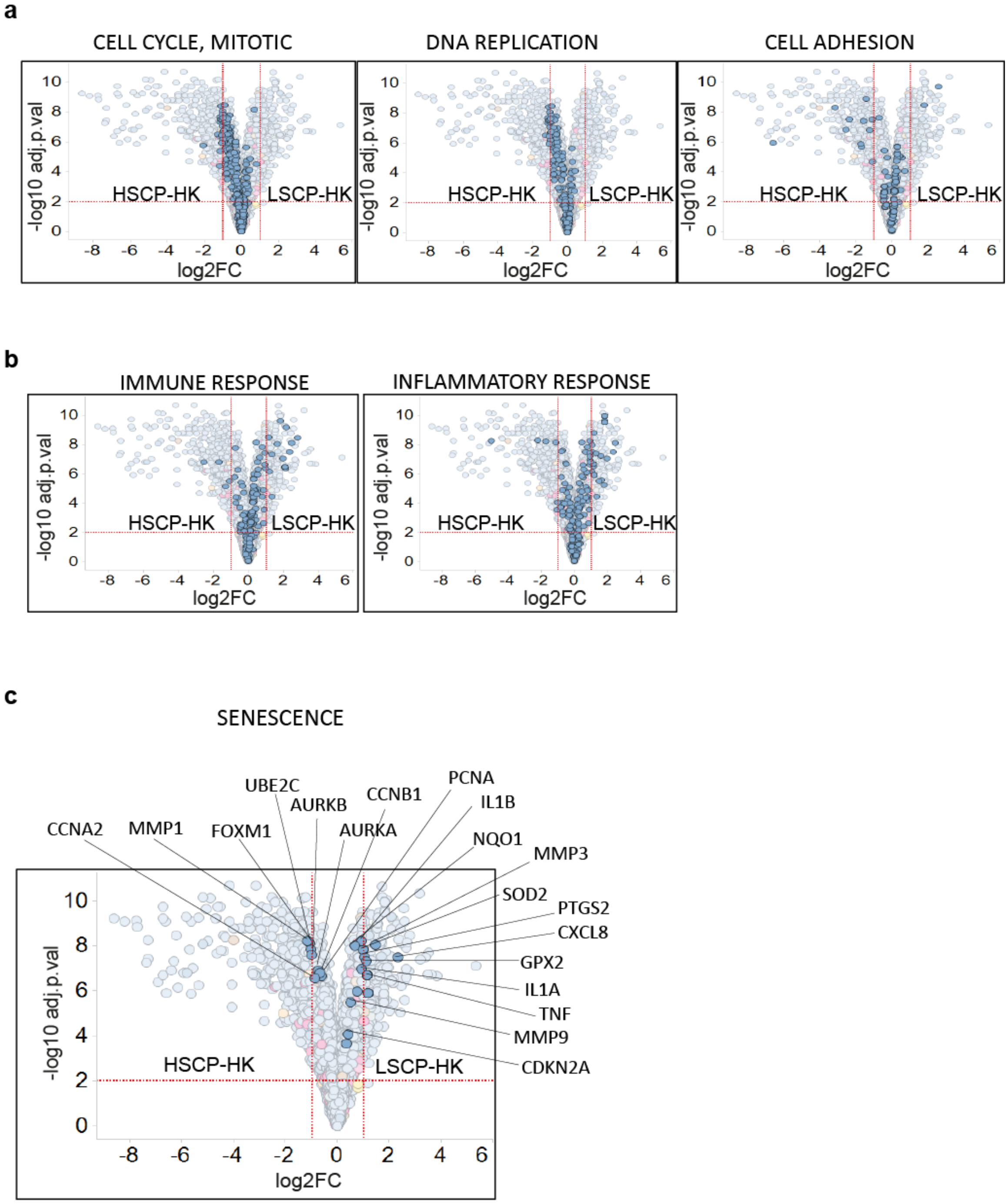

Supplementary Figure 1: Differential expression of GO term associated genes

Global difference in gene expression (RNA-seq) between HSCP-HKs and LSCP-HKs, plotting fold change (FC) versus adjusted p-value (adj.p.val). Dotted red lines indicate significance cut-offs defined as absolute value log2FC > 1 and -log10 adj.pval > 2. Genes associated with particular GO terms are highlighted in blue. (a) GO terms “Cell Cycle/Mitotic”, “DNA Replication” and “Cell Adhesion”. (b) GO terms “Immune Response” and “Inflammatory Response”. (c) Genes associated with senescence.

Supplementary Figure 2

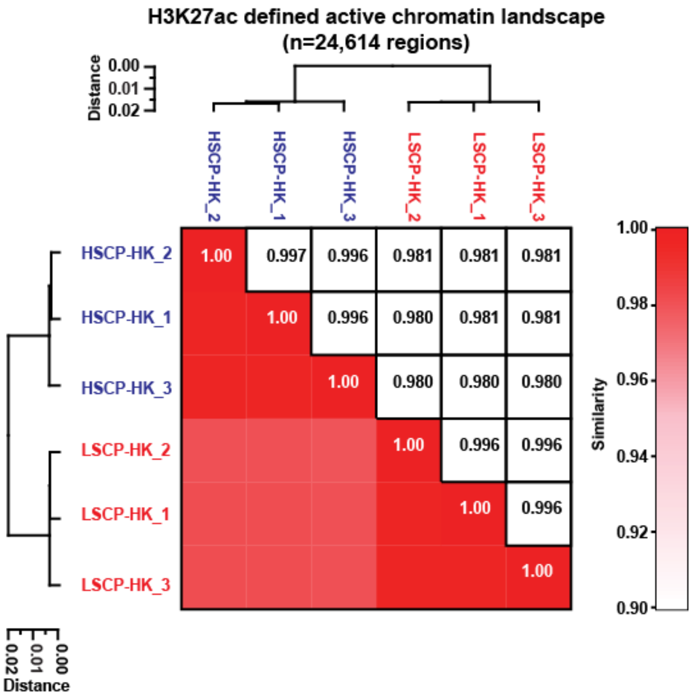

Supplementary Figure 2: H3K27ac distribution between HSCP-HKs and LSCP-HKs.

Pairwise similarity correlation of genome-wide H3K27ac occupancy at the union of all enriched regions between datasets (24,614 discrete regions). Similarity calculated by Pearson correlation of median normalized H3K27ac signal (r > 0.98).

Supplementary Figure 3

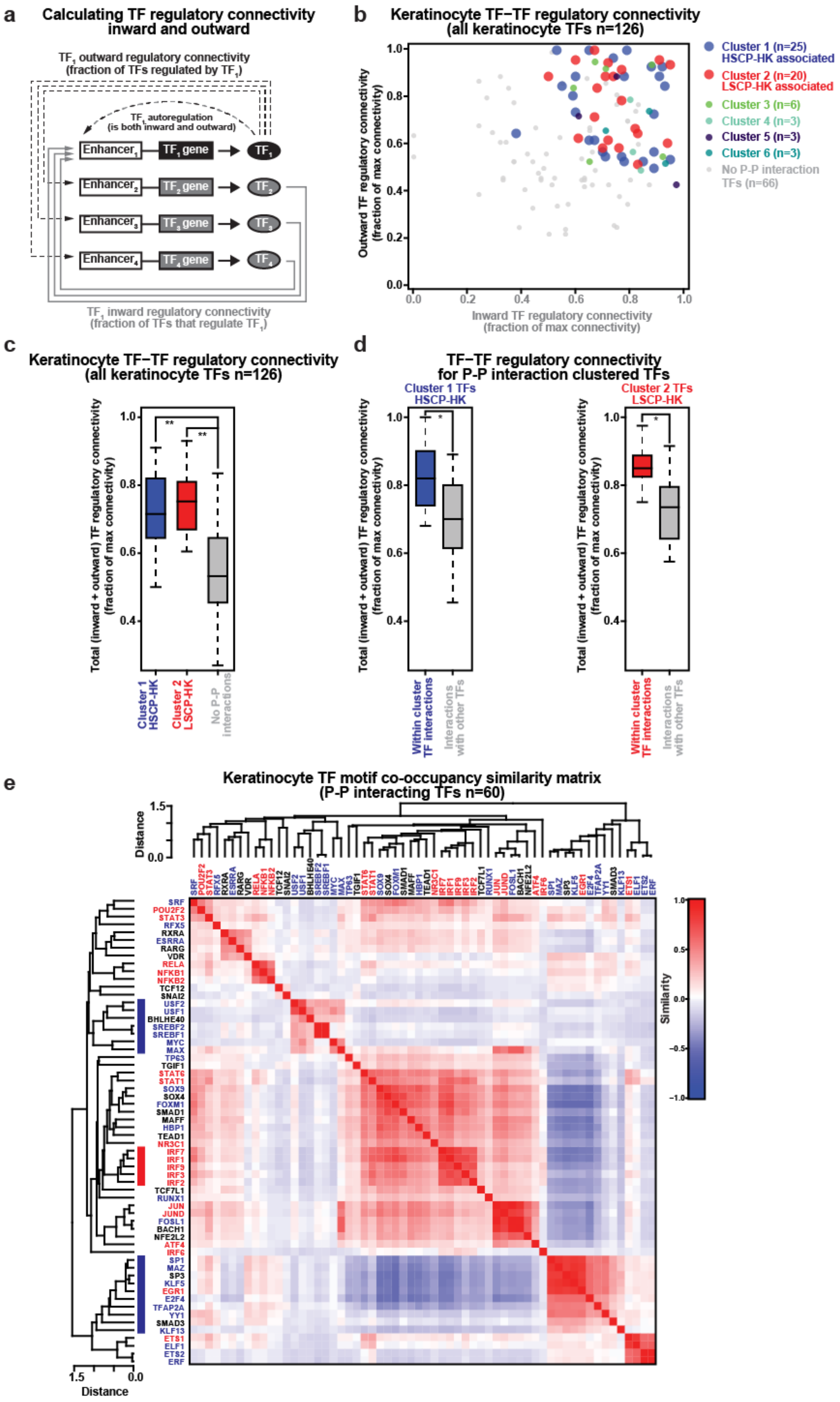

Supplementary Figure 3: Circuitry analysis identifies TF master regulators

(a) Schematic detailing how inward and outward regulatory connectivity is calculated for a given TF.

(b) Normalized inward (x-axis) and outward (y-axis) regulatory connectivity for all active and enhancer associated TFs in HSCP-HK and LSCP-HK (n=126). TFs are colored based on their cluster assignment by Markov Chain Linkage clustering.

(c) Total (inward + outward) normalized regulatory connectivity for TFs in cluster 1 (blue), cluster 2 (red), and TFs without annotated protein-protein interaction (grey). The statistical significance between distributions is shown by a Wilcox rank sum test (one sided). ** p-value < 1e-6. Box represents the 25-75 percentile, line represents the median, and whiskers extend 1.5x the 25-75 percentile range.

(d) Total (inward + outward) normalized regulatory connectivity for cluster 1 TFs (left, blue) and cluster 2 TFs (right, red). For each set of TFs, total normalized regulatory connectivity is shown for interactions within the cluster (left) and interactions with other active and enhancer associated TFs (right, grey). The statistical significance between distributions is shown by a Wilcox rank sum test (one sided). * p-value < 1e-3. Box represents the 25-75 percentile, line represents the median, and whiskers extend 1.5x the 25-75 percentile range.

(e) Pairwise similarity correlation of genome-wide TF motif occupancy for all CRC TFs (n=60). Similarity calculated by Pearson correlation of a binary motif occupancy matrix for all 49,986 discrete ATAC-seq regions predicted to harbor at least 1 CRC TF motif.

Supplementary Figure 4

a

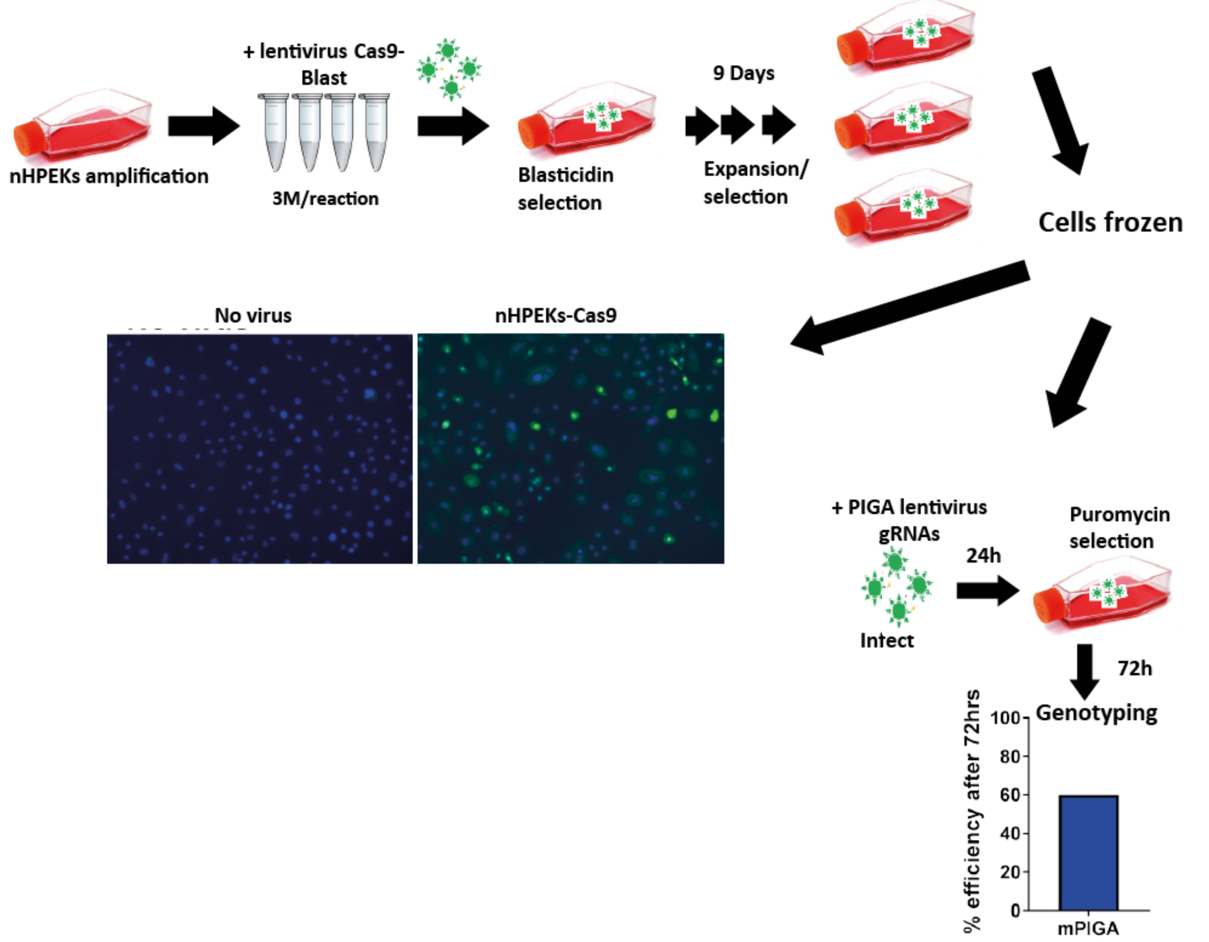

b

**IRF2**	**IRF6**	IRF3	RARG	**ETS1**	SMAD1	**SNAI2**	**JUND**	SRF	**POU2F2**
IRF9	**IRF1**	ELF1	**ESRRA**	STAT6	ATF4	**SOX4**	**TEAD1**	**FOSL1**	**TFAP2A**
**ERF**	**STAT1**	TCF7L1	HBP1	**FOXM1**	**NFKB1**	**NR3C1**	TCF12	**KLF5**	**MYC**
SREBE1	ETS2	**MAFF**	NFKB2	**TGIF1**	USF1	STAT3	**SMAD3**	SP3	**EGR1**
**IRF7**	RFX5	USF2	**RUNX1**	KLF13	**SOX9**	**NFE2L2**	MAX	SP1	**E2F4**
SREBE2	RXRA	**VDR**	**TP63**	**RELA**	**BHLHE40**	**JUND**	BACH1	**MAZ**	YY1

CRC TFs

- LSCP-HK associated TF
- HSCP-HK associated TF
- "Neutral" associated TF

TF: TF edited in the MiniPool CRISPR

Supplementary Figure 4: Generation of a Cas9-expressing primary keratinocyte cell line

(a) Schematic depicting the establishment of Cas9-expressing neonatal HPEKs (nHPEK-Cas9) and their expansion and cryopreservation. Cas9 expression was detected by immunocytochemistry using an anti-Cas9 antibody. nHPEK-Cas9 were assessed for editing efficiency against PIGA using the software TIDE.

(b) List of Core Regulatory Circuitry (CRC) TFs (from Fig. 3d) are displayed and shown as associated with a HSCP (blue), LSCP (red) or "neutral" (green). sgRNA targeting TFs shown *underlined* and in *bold* were included in the pooled screening library.

Supplementary Figure 5

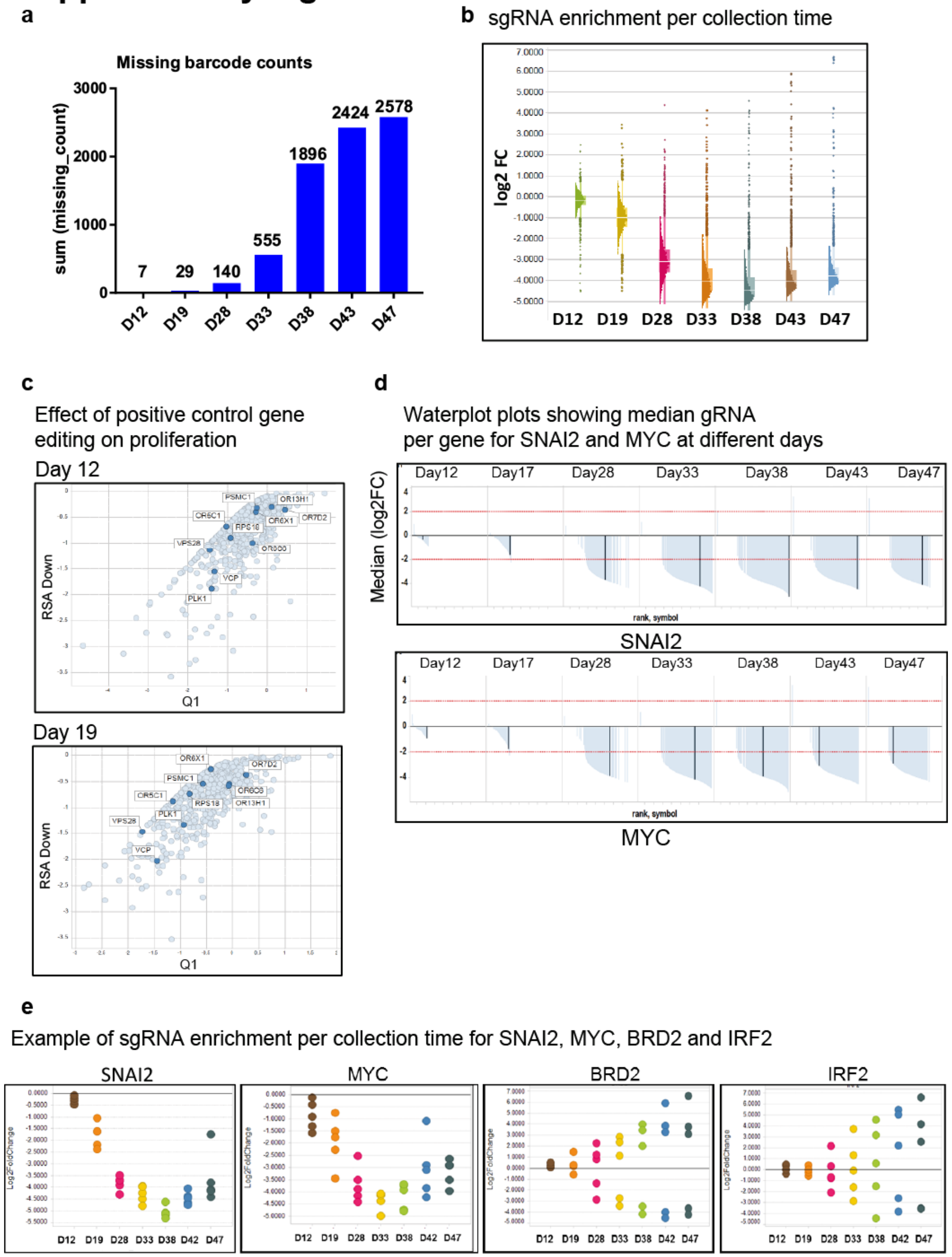

Supplementary Figure 5: CRISPR-Cas9 Screen results

(a) Missing sgRNA barcode counts over time from a total 2698 sgRNAs.

(b) sgRNA enrichment (fold change, FC) per collection time point.

(c) Effect of positive control gene editing (PLK1, VCP, OR6C6, VPS28, RPS18, OR5C1, OR6X1, OR7D2, OR13H1 and PSMC1) on proliferation at days 12 and 19.

(d) Median (log2FC) sgRNA per gene for SNAI2 and MYC per collection time point.

(e) Individual sgRNA enrichment (log2FC) per collection time point for SNAI2, MYC, BRD2 and IRF2, with 4 or 5 sgRNA per gene included in library at starting point.

Supplementary Figure 6

a

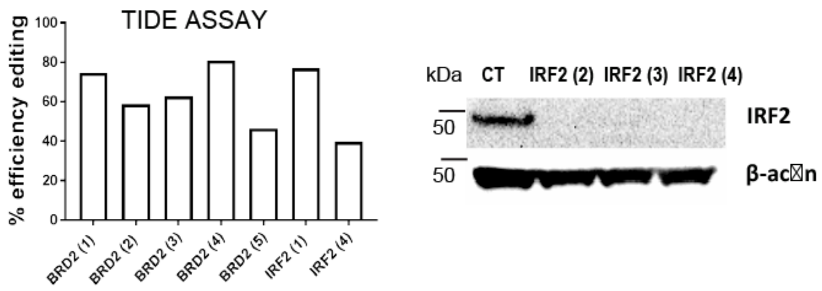

b

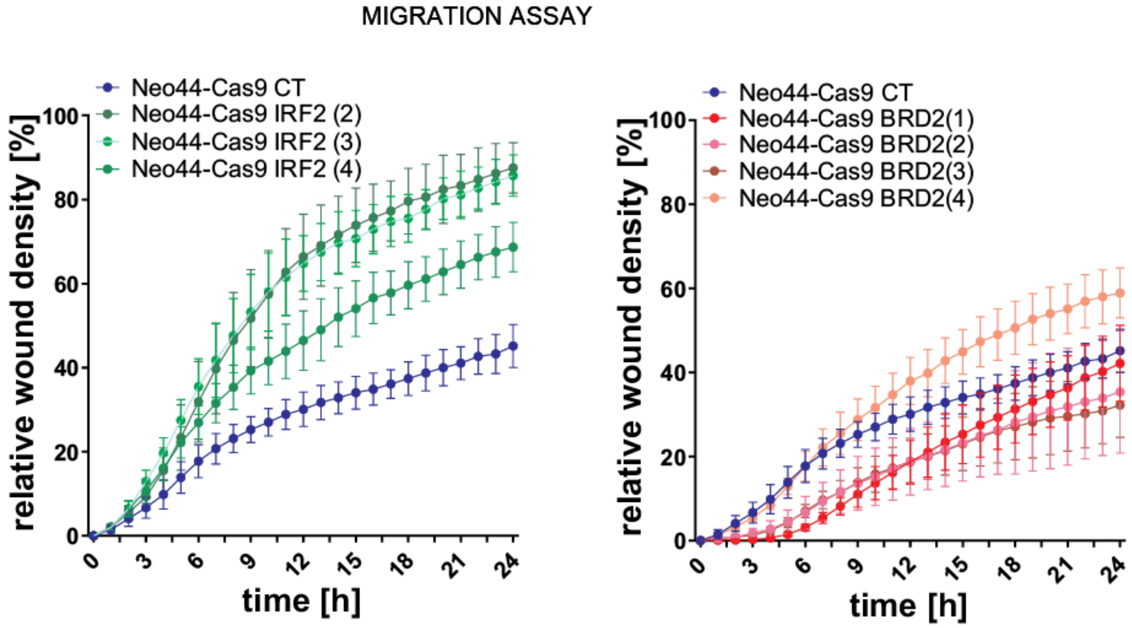

c

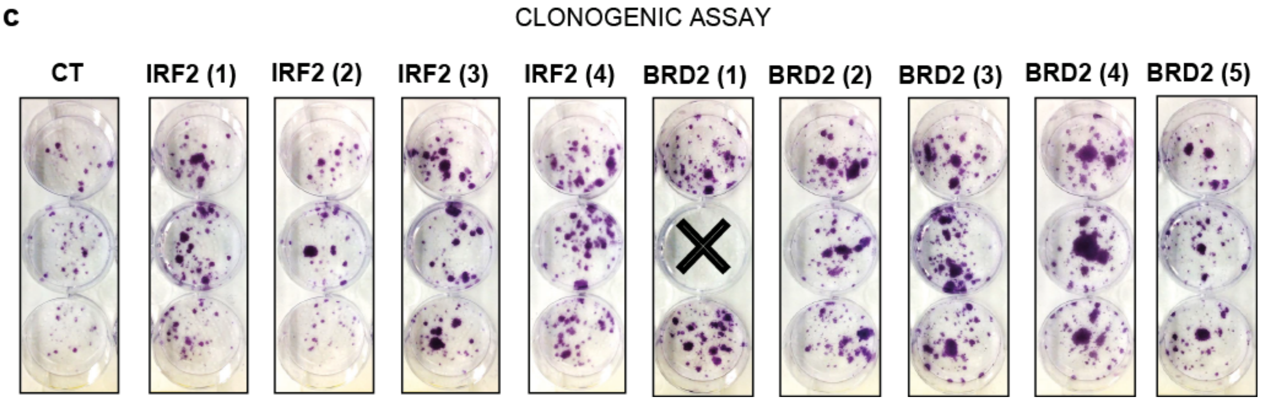

Supplementary Figure 6: Validation of individual sgRNAs for BRD2 and IRF2

(a) Individual lentivirus containing each of the five sgRNAs for BRD2 and four of IRF2 were used to infect nHPEK-Cas9s including a control sgRNA. Editing efficiency (in %) was quantified using the TIDE assay for BRD2 sg #1 to #5 and for IRF2 sg #1 and #4. Editing efficiency for IRF2 sg #2 to #4 was assessed at protein level by Western Blot.

(b) Migration assay with BRD2 and IRF2 edited nHPEK-Cas9.

(c) Clonogenic assay using 2000 cells per well in 6-well plates (in triplicates) comparing BRD2 and IRF2 -edited nHPEK-Cas9 with control cells (CT).

Supplementary Figure 7

a

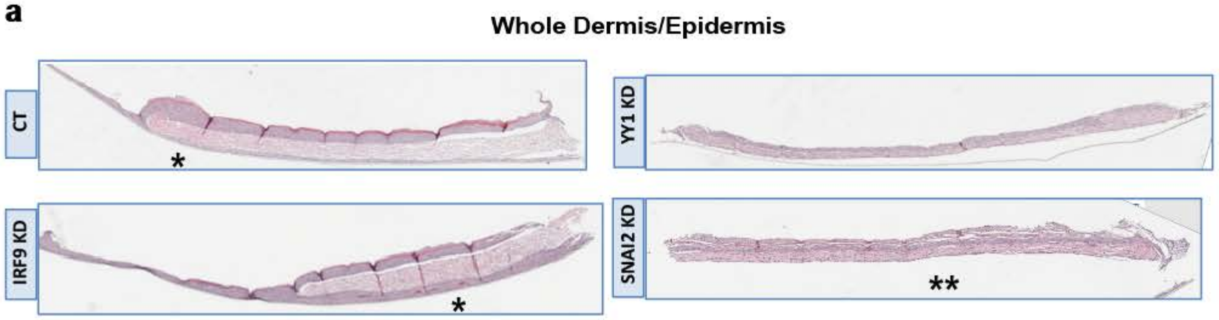

b

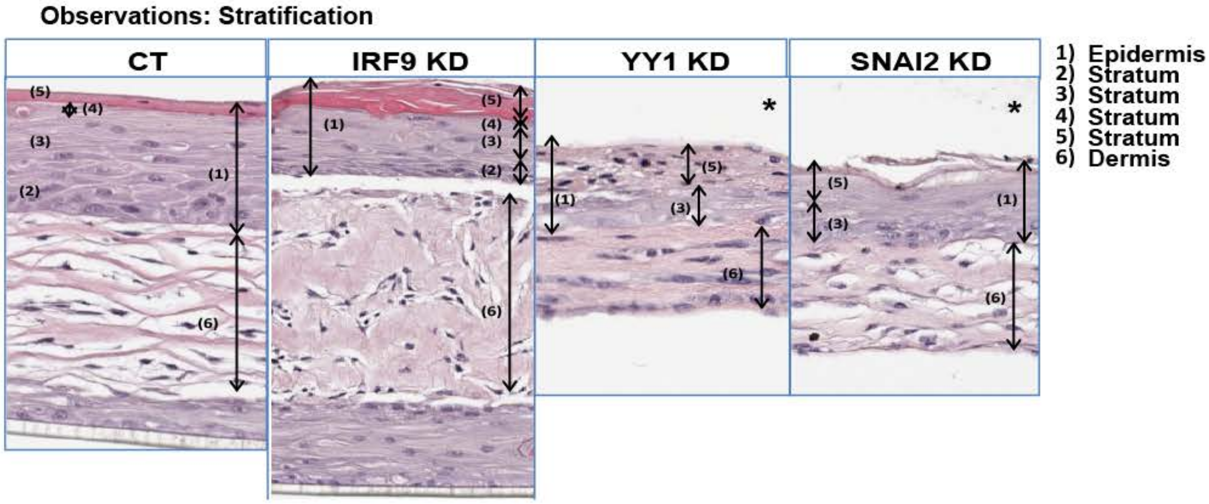

c

Effect of IRF9 editing in keratinocytes: clonogenic and migration

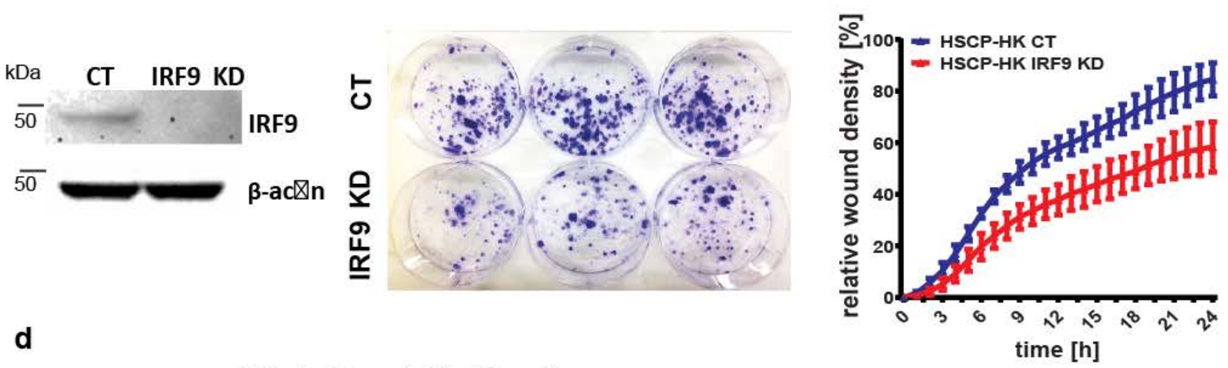

d

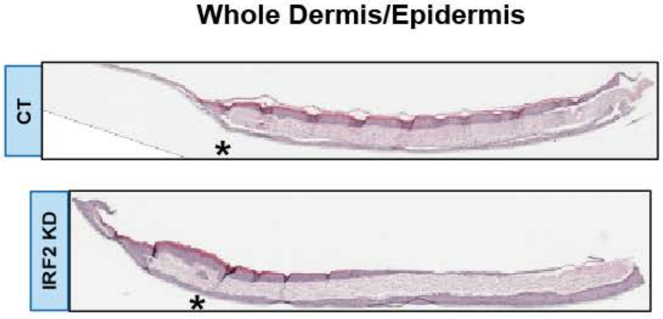

Supplementary Figure 7: Effect of loss of SNAI2, YY1, IRF9 or IRF2 on epidermis formation

(a) and (d) Epidermis formation by SNAI2 KD, IRF9 KD, YY1 KD, IRF2 KD or control cells (CT) in human dermo-epidermal 3D model. * Epidermis growth beneath the dermis. ** Minimal epidermis growth beneath the dermis.

(b) Stratification observed in human dermo-epidermal 3D model of epidermis generated by SNAI2 KD, IRF9 KD, YY1 KD or CT HSCP-HKs. Layers of human skin labeled as 1) to 6). * No clear stratification.

(c) IRF9 protein expression analysis by Western blot in aHPEKs IRF9 CRISPR-Cas9 KD cells versus control cells (CT) with β-actin as a loading control (left panel). Clonogenic assay (middle panel) and migration assay (right panel) comparing HSCP-HK IRF9 KD versus CT.

Supplementary Figure 8

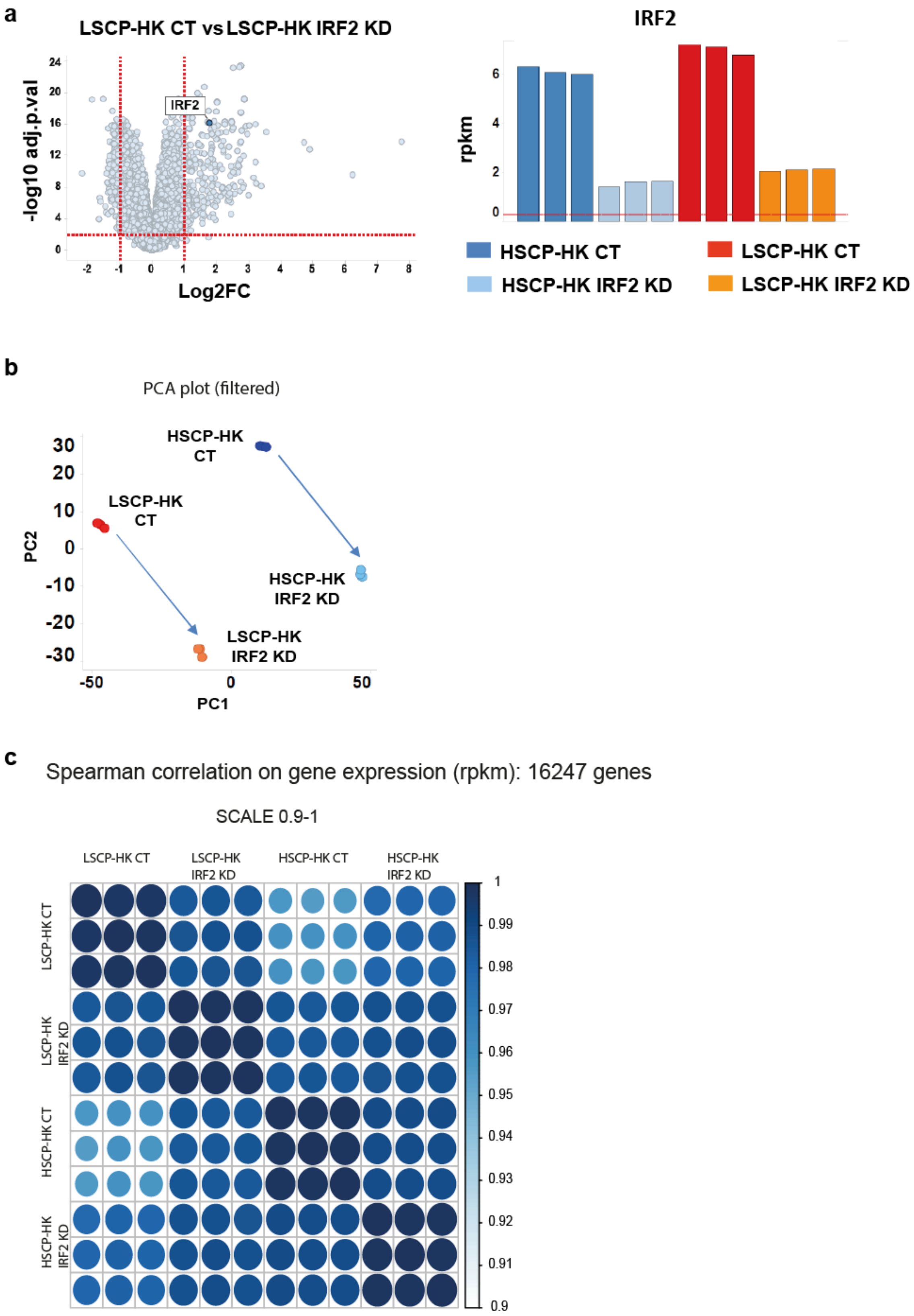

Supplementary Figure 8: Effect of IRF2 KD on global gene expression

(a) Global difference in gene expression (RNA-seq) between LSCP-HKs and LSCP-HKs with IRF2 KD, plotting fold change (FC) versus adjusted p-value (adj.p.val). Dotted red lines indicate significance cut-offs defined as absolute value log2FC > 1 and -log10 adj.pval > 2. IRF2 gene highlighted in blue (left panel).IRF2 transcript level based on RNA-seq (rpkm) across conditions and individual replicates (right panel)

(b) Principal component analysis (PCA) and (c) Spearman correlation (scaled 0.9-1) of gene expression (rpkm) across the 4 conditions.

Supplementary Figure 9

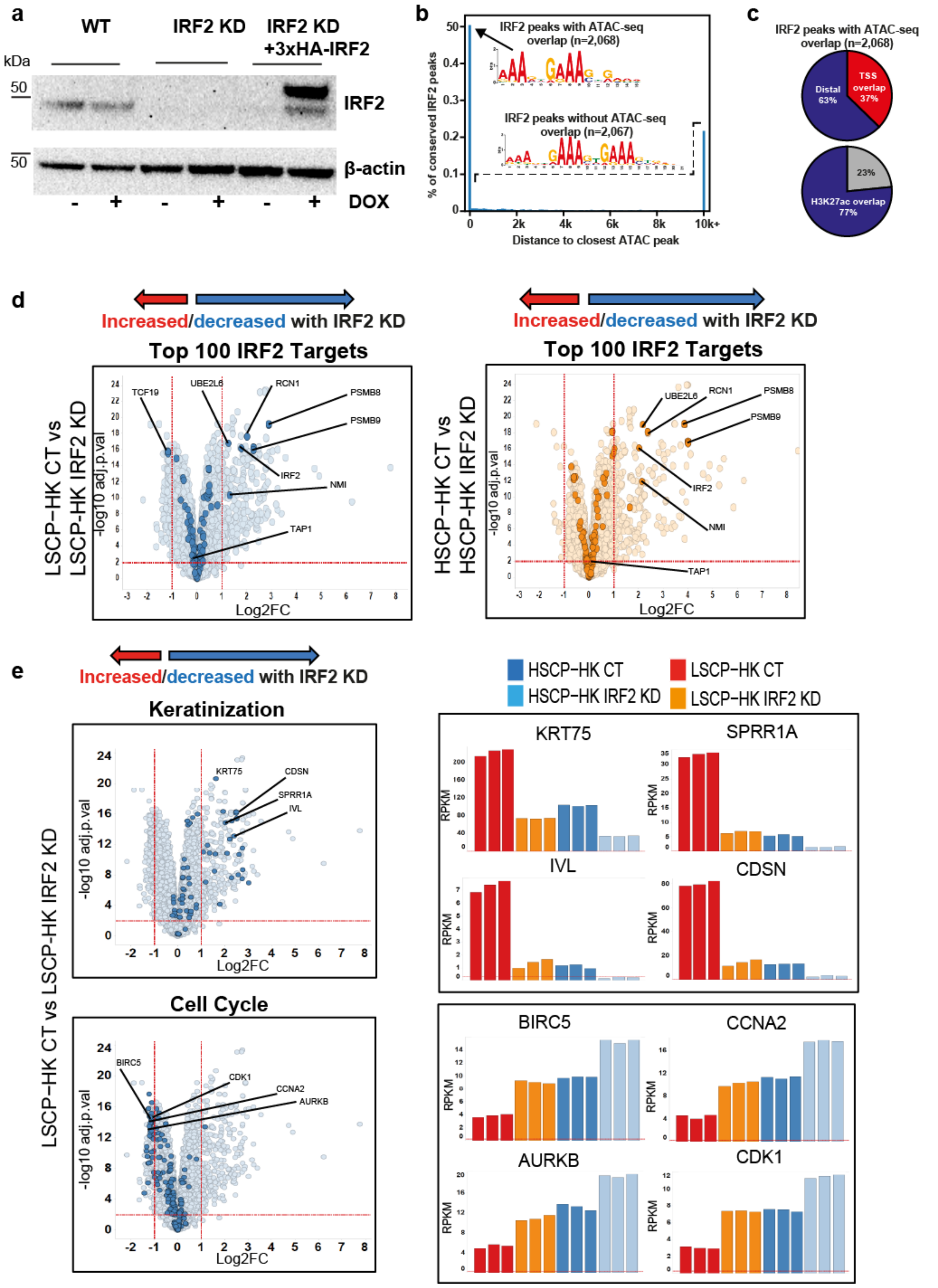

Supplementary Figure 9: Effect of IRF2 KD on its direct and indirect target genes

(a) IRF2 protein level analysis by Western blot in aHPEKs wild type (WT), IRF2 CRISPR-Cas9 KD or IRF2 CRISPR-Cas9 KD plus 3xHA-IRF2 overexpressing cells in the absence or presence of 5 day treatment with doxycylcine, using β-actin as a loading control.

(b) Histogram of IRF2 ChIP peaks showing the distance to the nearest ATAC peak. Insets show the de novo top motif found by MEME for either IRF2 ChIP peaks overlapping an ATAC peak (left) or those >10kb away from an ATAC peak.

(c) For IRF2 ChIP peaks that overlap ATAC peaks (n=2,072), pie charts showing overlap of IRF2 peaks with left: gene transcription start sites (TSS) or right: H3K27ac peaks

(d) Global difference in gene expression (RNA-seq) upon IRF2 KD in LSCP-HKs (left panel) or HSCP-HKs (right panel), plotting fold change (FC) versus adjusted p-value (adj.p.val). Dotted red lines indicate significance cut-offs defined as absolute value log2FC > 1 and -log10 adj.pval > 2. Top 100 IRF2 target genes are highlighted.

(e) Same as (d) left panel, but highlighting “Keratinization” (top panel) and “Cell Cycle” (bottom panel) associated genes. Keratinization (Biosystems, -log10 adj.pval= 26) genes are mainly downregulated (cluster 1 of heatmap) and Cell Cycle genes (Biosystems, -log10 adj.pval= 16.7) are mainly upregulated (cluster 2 in heatmap) in LSCP-HK IRF2 KD vs CT. Examples of individual genes highly differentially expressed between HSCP-HKs and LSCP-HKs and for which expression can be restored to levels of HSPC-HK by IRF2 KD in LSCP-HK from either the keratinization cluster (KRT75, SPRR1A, IVL and CDSN) or the cell cycle cluster (BIRC5, CCNA2, AURKB, CDK1) are shown at the transcript level (rpkm) across conditions and replicates.

Supplementary Figure 10

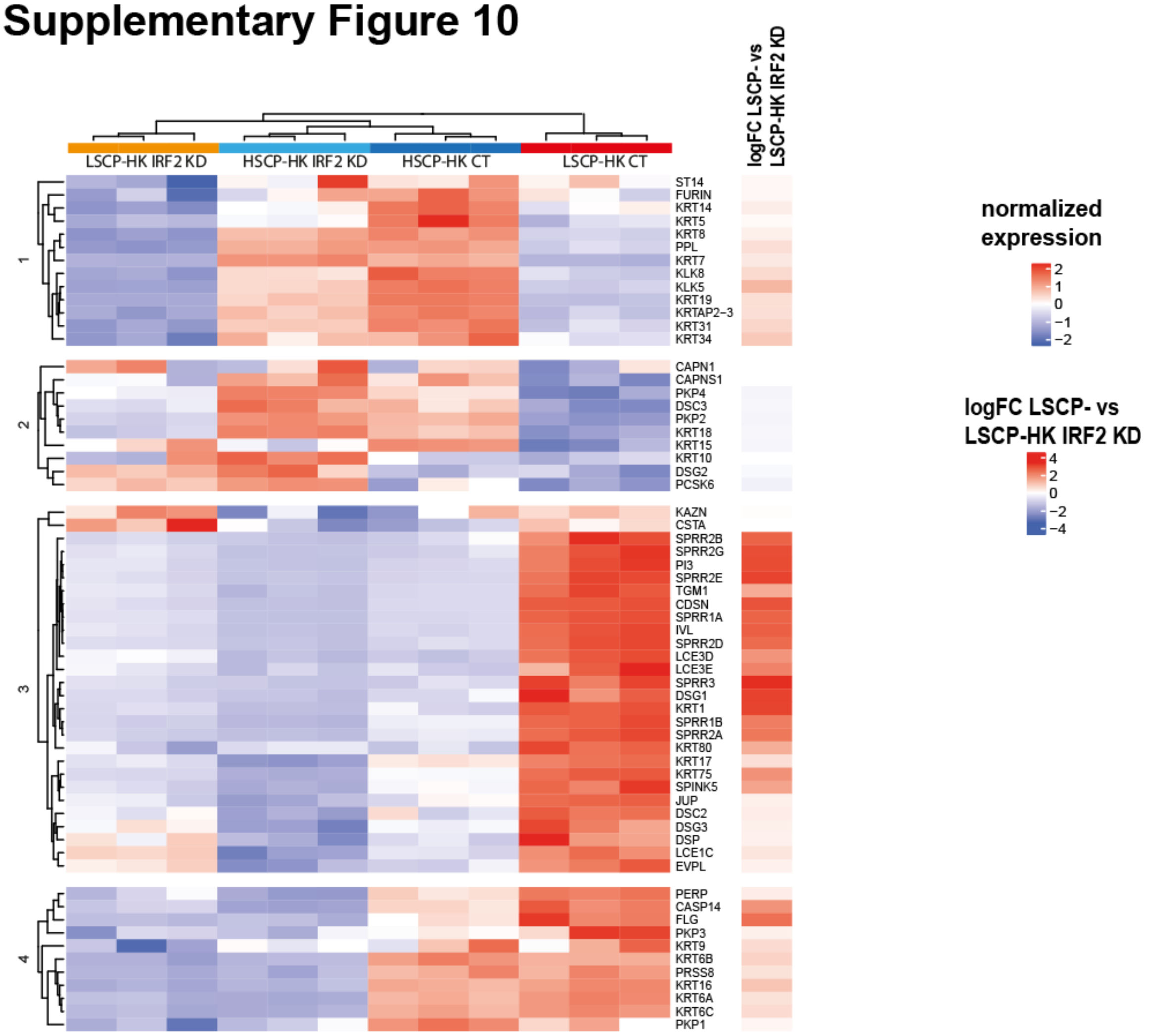

- Similar as genome-wide: IRF2 KO in LSCP-HK clusters with HSCP-HK samples
- Cluster 3 shows enrichment for Keratinization genes that are involved in **Keratinocyte Differentiation**

Cluster	Interpretation:	GO:Keratinocyte differentiation	GO: Cornification	GO: Keratinization
1	genes are still higher in HSCP than LSCP-HK after IRF2 KD		TOP 1 (23.64)	TOP 2 (8.94)
2	genes that go up by IRF2 KD in LSCP and HSCP		TOP 1 (19.2)	TOP 2 (9.32)
3	genes that go down and back to HSCP-HK in LSCP-HK IRF2 KD	TOP 3 (31.99)	TOP 1 (56.36)	TOP 5 (14.81)
4	genes that go down by IRF2 KD in LSCP and HSCP	TOP 6 (2.28)	TOP 1 (19.37)	TOP 2 (9.82)

Supplementary Figure 10: IRF2 KD in LSCP-HK downregulates a Keratinization-associated expression program

Hierarchical clustering of Keratinization-associated genes based on comparison of gene expression (rpkm) upon IRF2 KD in HSCP-HK or LSCP-HK. Four clusters are shown and annotated in table at the bottom. Fold change gene expression changes between LSCP-HK CT and IRF2 KD for individual genes per cluster are depicted right of the main heat map. Cluster annotation table lists intersection with GO terms “Keratinocyte Differentiation”, “Cornification” and “Keratinization” plus respective p-value in brackets.

Supplementary Figure 11

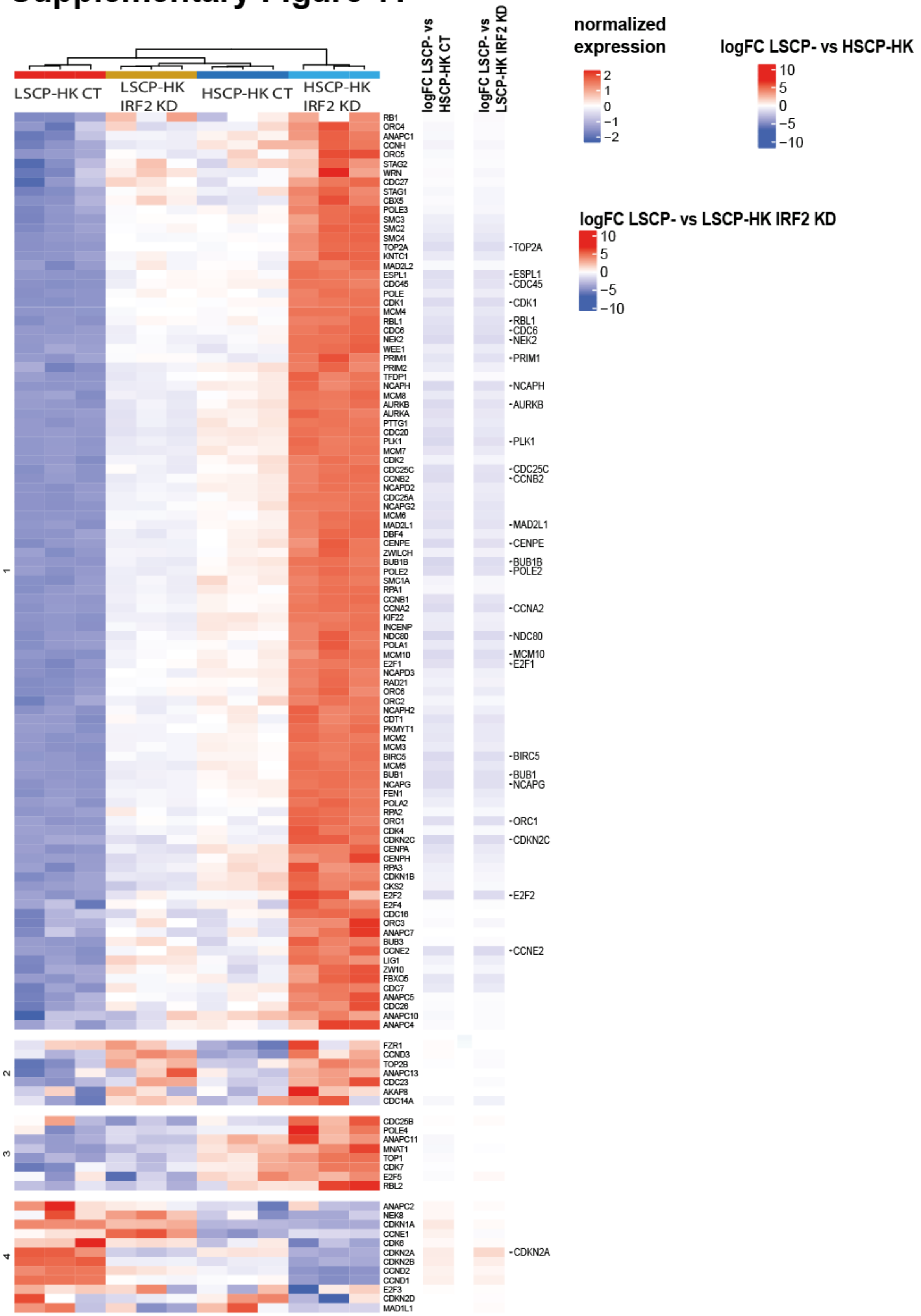

Supplementary Figure 11: IRF2 KD in HSCP-HK upregulates a Cell Cycle associated expression program

Hierarchical clustering of Cell Cycle-associated genes based on comparison of gene expression (rpkm) upon IRF2 KD in HSCP-HK or LSCP-HK. Four clusters are shown with individual genes per cluster listed. Fold change gene expression changes between either LSCP-HK versus HSCP-HKs or LSCP-HK CT versus IRF2 KD for individual genes per cluster are depicted in the two panels to the right.

Supplementary Figure 12

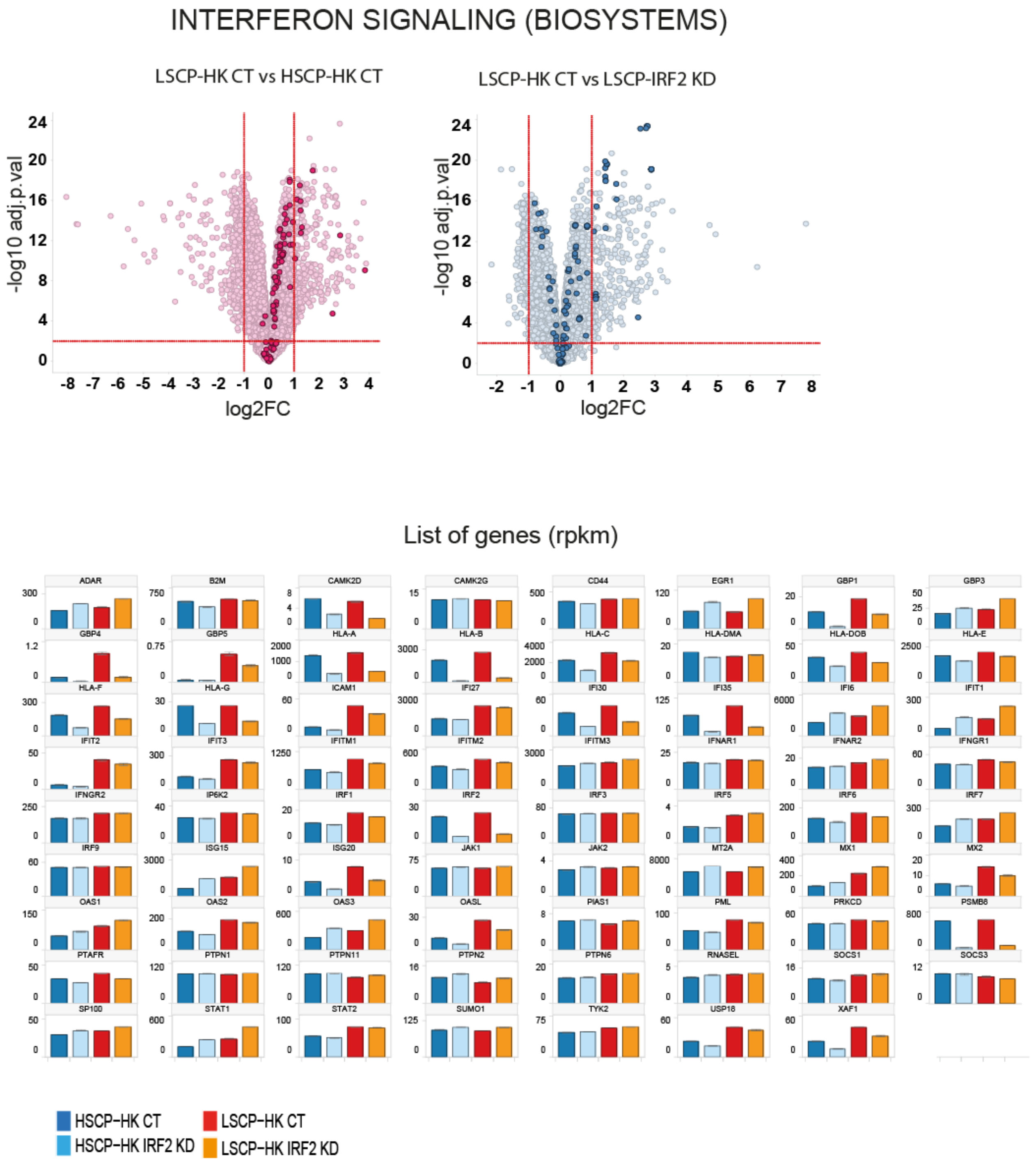

Supplementary Figure 12: Effect of IRF2 KD on Interferon signaling

Global difference in gene expression (RNA-seq) between LSCP-HKs versus HSCP-HKs (left panel) or upon IRF2 KD in LSCP-HKs (right panel), plotting fold change (FC) versus adjusted p-value (adj.p.val). Dotted red lines indicate significance cut-offs defined as absolute value log2FC > 1 and -log10 adj.pval > 2. Genes associated with GO term “Interferon Signaling” (Biosystems -log10 adj.pval= 16.2) are highlighted. Transcript level expression by RNA-seq (rpkm) across conditions for all genes represented in Interferon Signaling GO group are shown in bottom panel.

Supplementary Figure 13

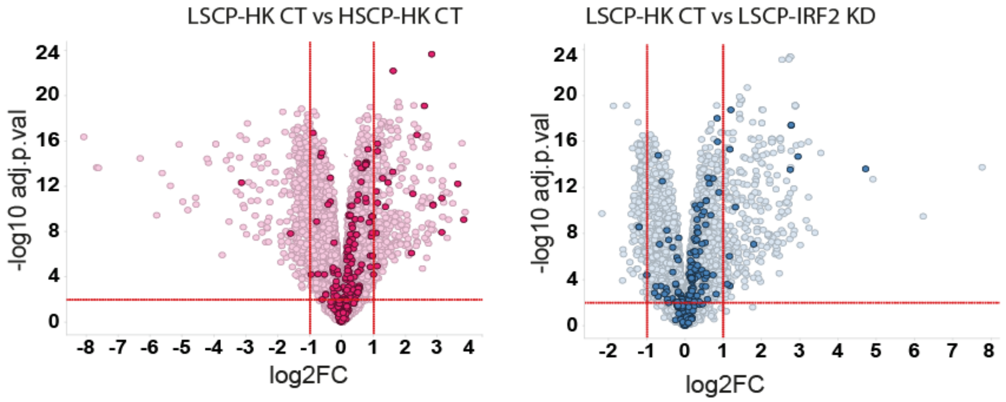

List of genes (rpkm)

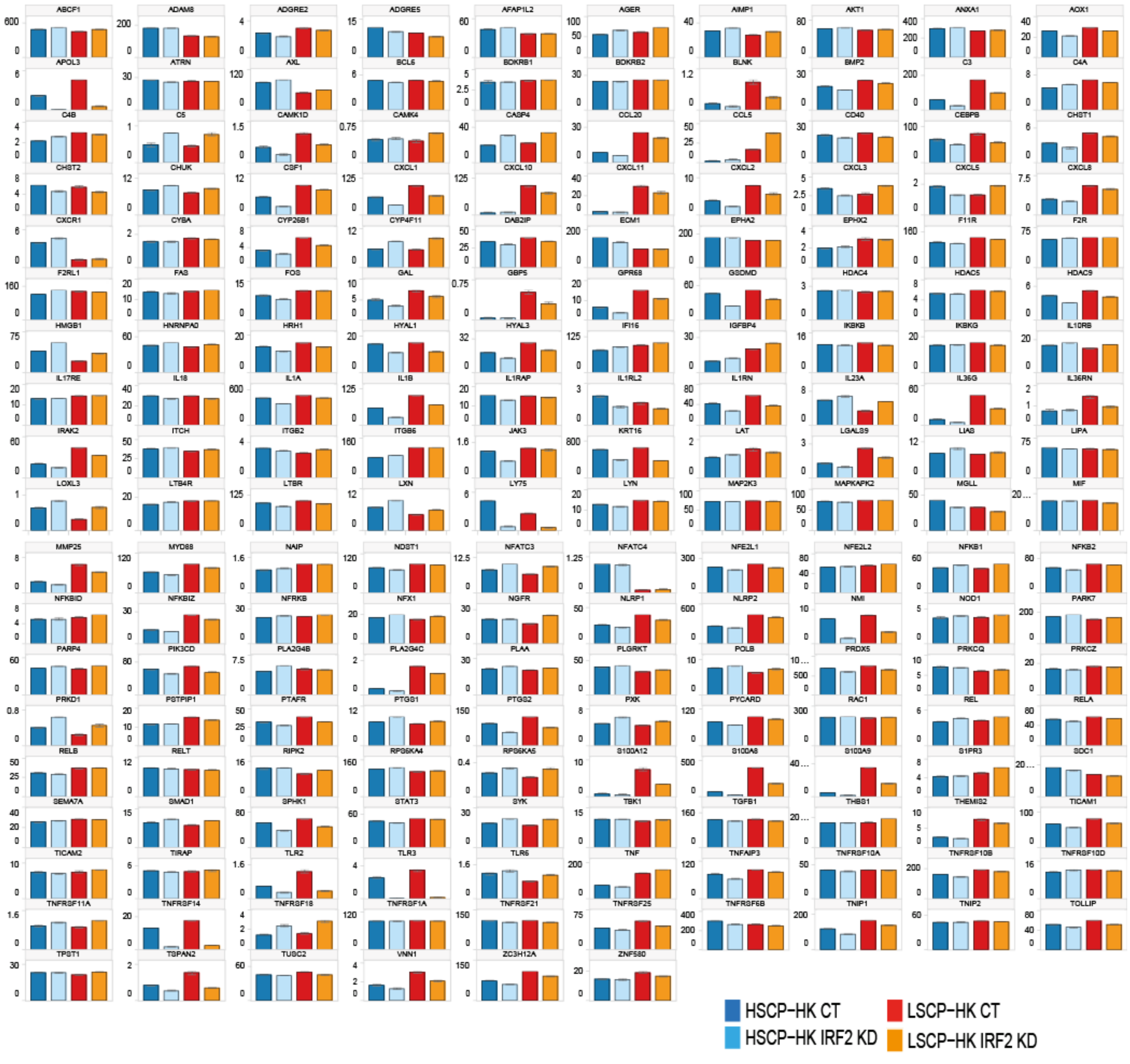

Supplementary Figure 13: Effect of IRF2 KD on Inflammatory Response

Global difference in gene expression (RNA-seq) between LSCP-HKs versus HSCP-HKs (top panel) or upon IRF2 KD in LSCP-HKs (bottom panel), plotting fold change (FC) versus adjusted p-value (adj.p.val). Dotted red lines indicate significance cut-offs defined as absolute value log2FC > 1 and -log10 adj.pval > 2. Genes associated with GO term "Inflammatory Response" (Biosystems -log10 adj.pval= 17.4) are highlighted. Transcript level expression by RNA-seq (rpkm) across conditions for all genes represented in Inflammatory Response GO group are shown in bottom panel.

Supplementary Figure 14

PSORIASIS STUDY 14/ NORMAL SKIN TISSUE (genevestigator)

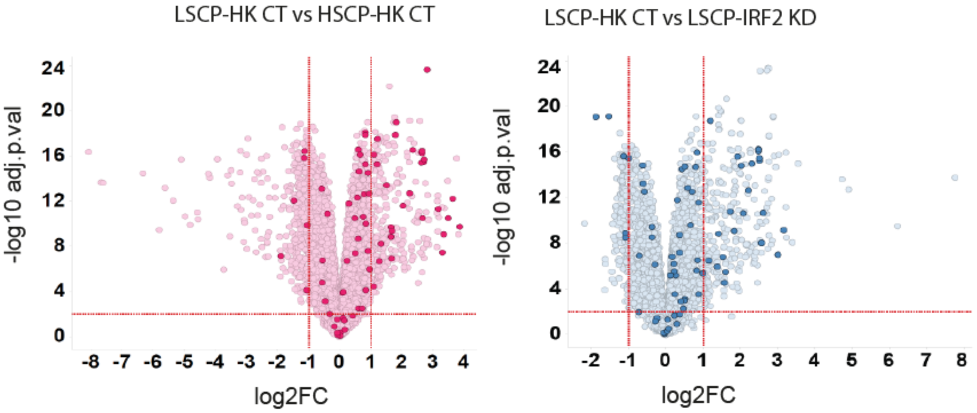

List of genes (rpkm)

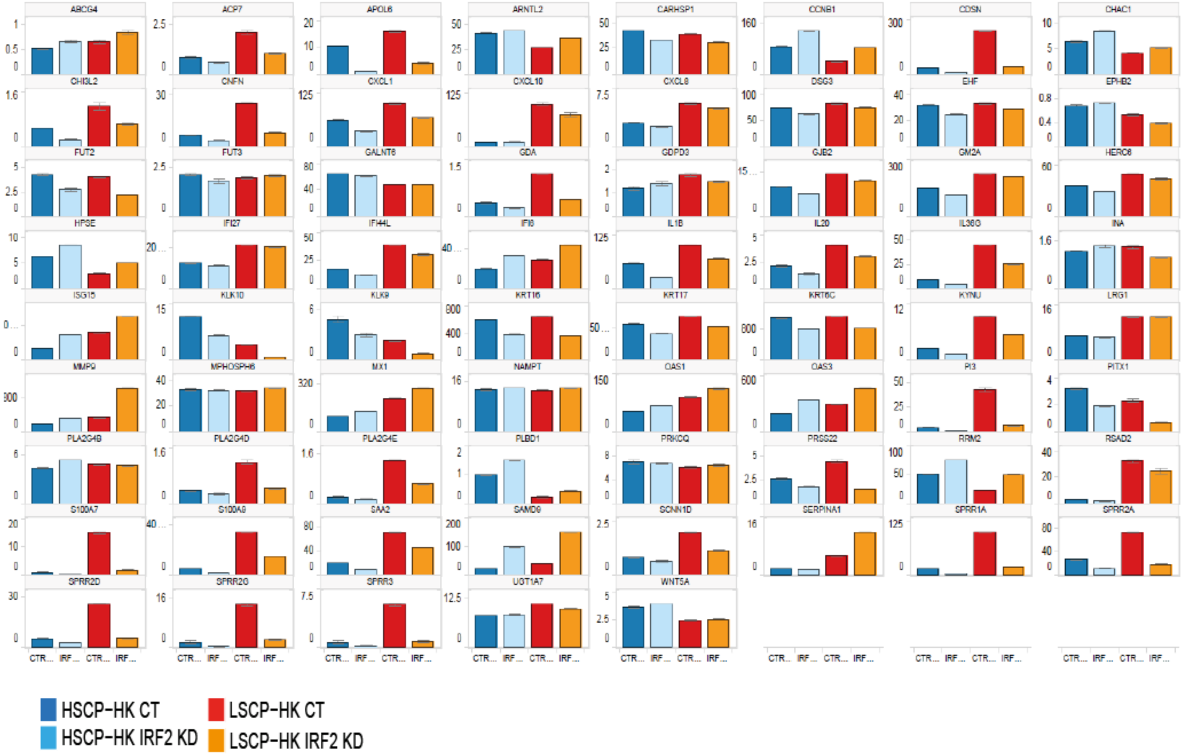

Supplementary Figure 14: Effect of IRF2 KD on Psoriasis-associated genes

Global difference in gene expression (RNA-seq) between LSCP-HKs versus HSCP-HKs (left panel) or upon IRF2 KD in LSCP-HKs (right panel), plotting fold change (FC) versus adjusted p-value (adj.p.val). Dotted red lines indicate significance cut-offs defined as absolute value log2FC > 1 and -log10 adj.pval > 2. Genes associated with GO term “PSORIASIS STUDY 14/NORMAL SKIN TISSUE” (Genevestigator (Hruz et al., 2008). Comparison LSCP-HKs to HSCP-HKs: -log10 adj.pval = 29.6. Comparison LSCP-HKs CT to LSCP-HKs IRF2 KD: -log10 adj.pval= 21.3. Transcript level expression by RNA-seq (rpkm) across conditions for all genes represented in PSORIASIS STUDY 14/NORMAL SKIN TISSUE are shown in bottom panel.

Supplementary Figure 15

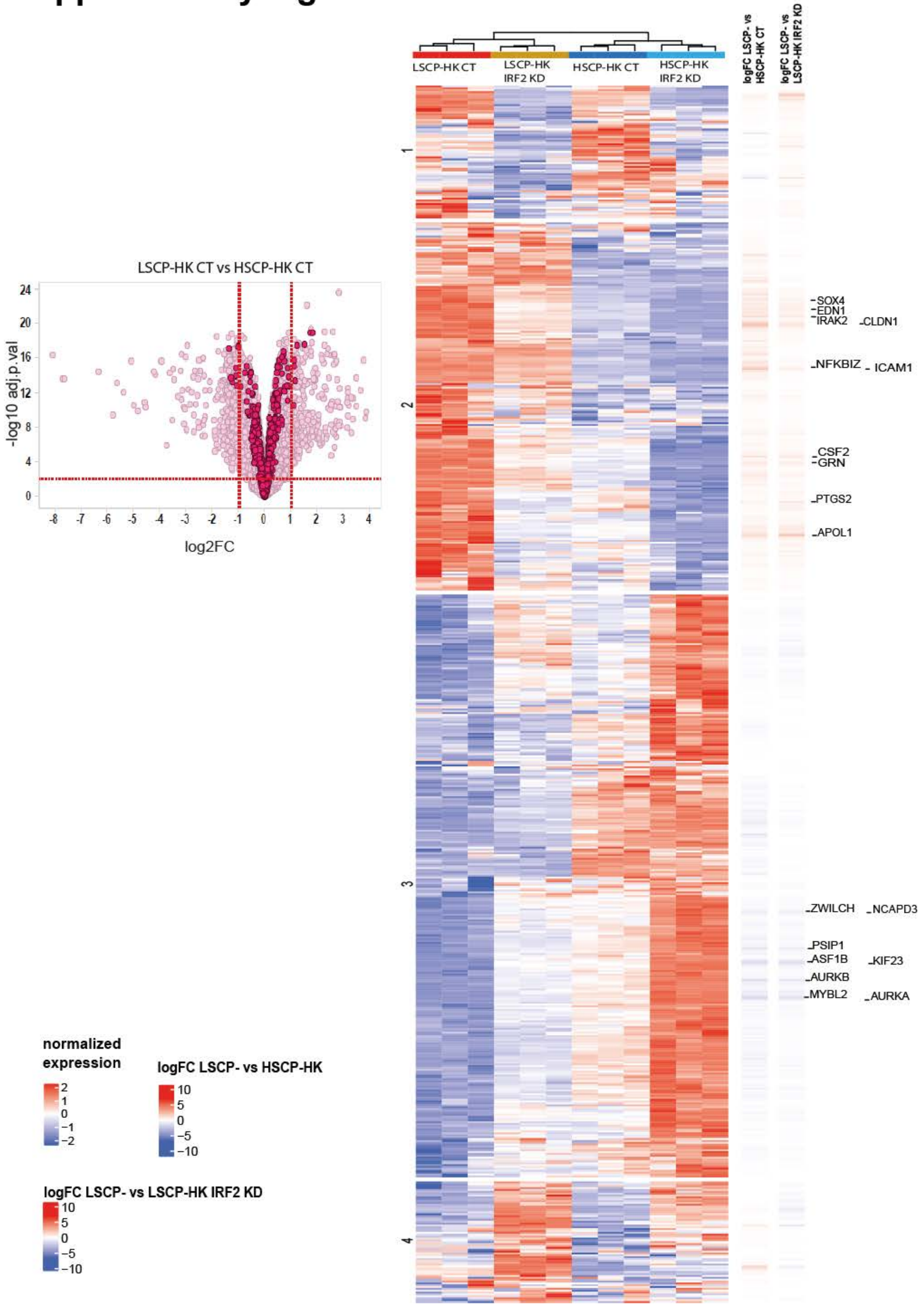

Supplementary Figure 15: Genes with increased IRF2 activity in their regulatory regions

(Left panel) Global difference in gene expression (RNA-seq) between LSCP-HKs versus HSCP-HKs, plotting fold change (FC) versus adjusted p-value (adj.p.val). Dotted red lines indicate significance cut-offs defined as absolute value log2FC > 1 and -log10 adj.pval > 2. Genes directly regulated by IRF2 (Table S6) with increased IRF2 activity in their regulatory regions in LSCP-HK compared to HSCP-HK (from Fig. 3g) are highlighted in red.

(Middle panel) Hierarchical clustering of genes associated with predicted increased IRF2 in their regulatory region (Fig. 3d) in LSCP-HK vs HSCP-HK based on comparison of gene expression (rpkm) upon IRF2 KD in HSCP-HK or LSCP-HK. Four clusters are shown.

(Right panel) Fold change gene expression changes between either LSCP-HK versus HSCP-HKs or LSCP-HK CT versus IRF2 KD for individual genes per cluster are depicted. Highlighted genes indicate genes with differential expression in LSCP-HK vs HSCP-HK, respectively.

Supplementary Figure 16

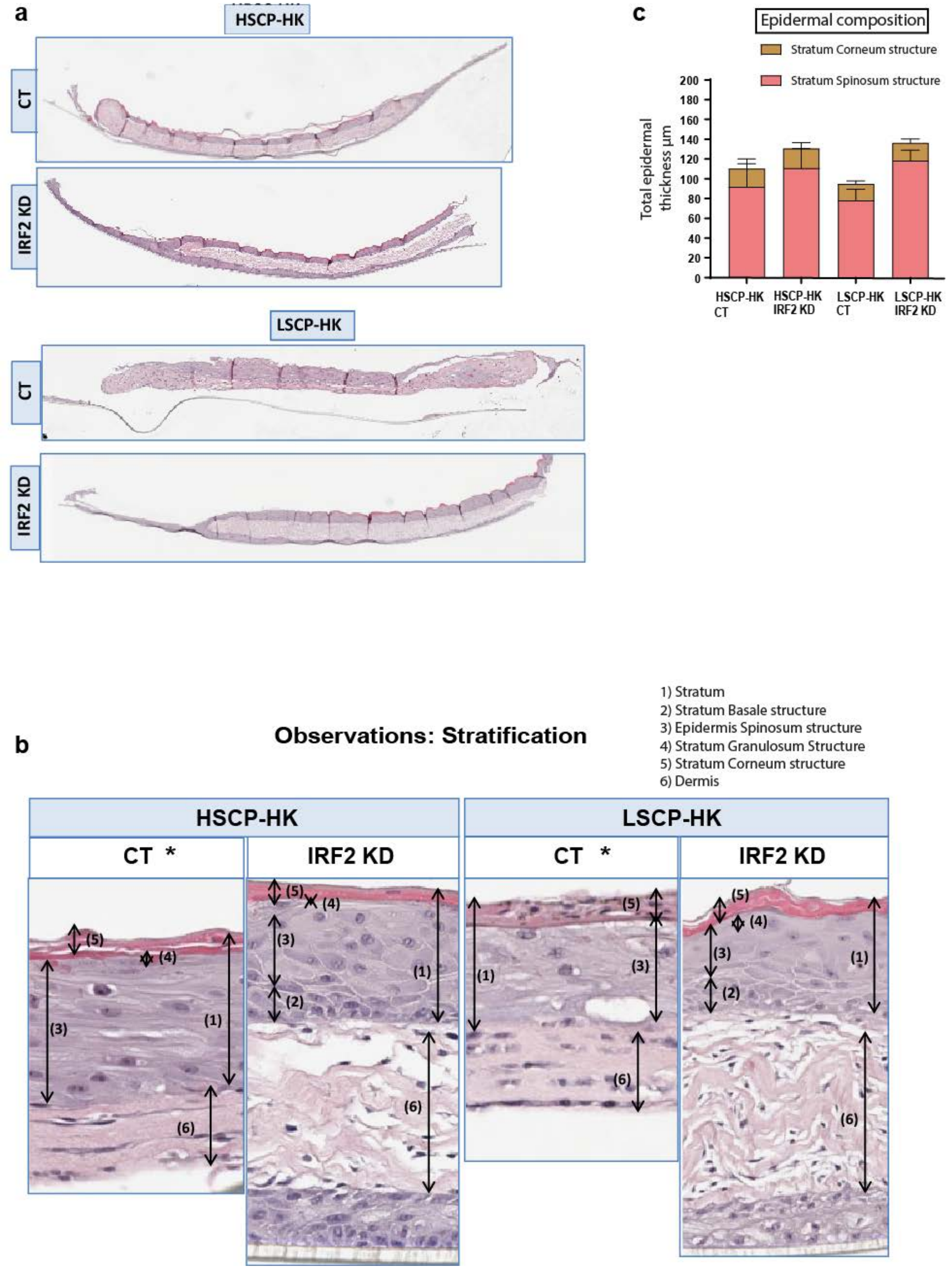

Supplementary Figure 16: Effect of IRF2 KD on epidermis development

(a) Epidermis generation in human dermo-epidermal 3D model comparing IRF2 KD in HSCP-HK versus LSCP-HKs.

(b) Stratification observed in human dermo-epidermal 3D model upon IRF2 KD in HSCP-HKs versus LSCP-HKs. Layers 1) to 6) of human skin indicated. * No clear separation of Stratum Basale structure.

(c) Quantification of epidermal composition via Stratum Corneum and Stratum Spinosum structures generated upon IRF2 KD in HSCP-HKs versus LSCP-HKs. Thickness (µm) was quantified for each condition (means ± S.D. of n=4 biological replicates).

Supplementary Figure 17

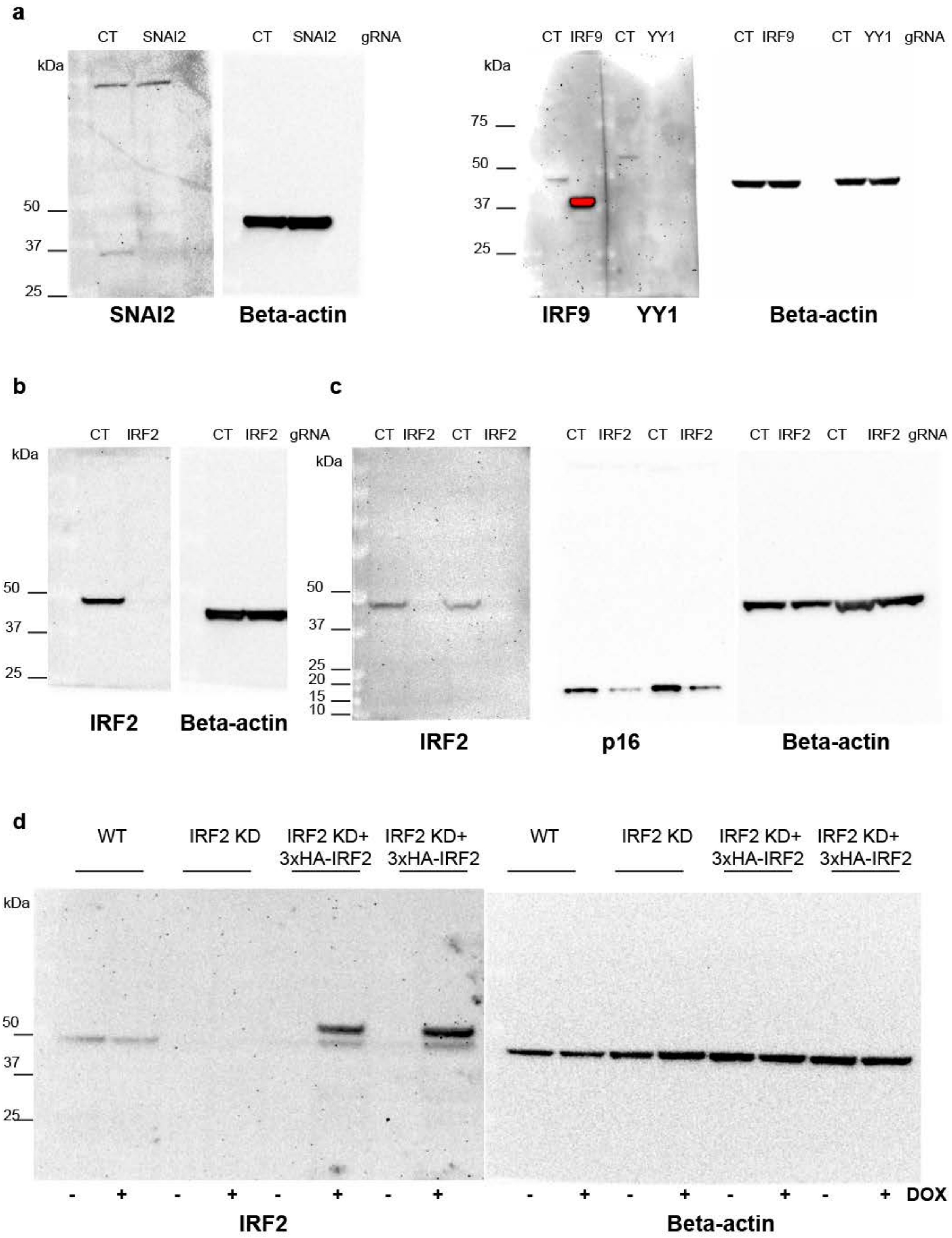

Supplementary Figure 17: Whole Western blot pictures

(a) CRISPR-Cas9 mediated knock-down (KD) of SNAI2 (left panel) or YY1 and IRF9 (right panel) protein in HSCP-HK monitored by Western blot analysis utilizing β-actin protein level as a loading control (expected MW: 45kDa). Left for each blot, MW indicated in kDa. SNAI2 expected MW: 30kDa. YY1 expected MW: 65kDa and IRF9 expected MW: 48kDa.

(b) CRISPR-Cas9 mediated knock-down (KD) of IRF2 protein in HSCP-HK monitored by Western blot analysis utilizing β-actin protein level as a loading control (expected MW: 45kDa). Left of the blots, MW indicated in kDa. IRF2 expected MW: 50kDa.

(c) Effect of CRISPR-Cas9 KD of IRF2 in HSCP-HK and LSCP-HK cells on IRF2 (expected MW: 50kDa) and p16 INK4A (expected MW: 16kDa) protein expression as measured by Western blot using β-actin as a loading control (expected MW: 45kDa). Left of the blots, MW indicated in kDa.

(d) IRF2 protein level analysis by Western blot in aHPEKs wild type (WT), IRF2 CRISPR-Cas9 KD or IRF2 CRISPR-Cas9 KD plus 3xHA-IRF2 (shown twice) overexpressing cells in the absence or presence of 5 day treatment with doxycycline, using β-actin as a loading control (expected MW: 45 kDa). Left of the blots, MW indicated in kDa.

Supplementary Tables

Supplementary Table 1

IRF2

gRNA1: antisense in exon3: GGATGCATGCGGCTAGACAT

gRNA2: antisense intron3-4: CCTGCGGCGTTTATAGAGGA

Generate -/+ 87bp excision

SNAI2

gRNA1: sense in exon2: GTAACTCTCATAGAGATACG

gRNA2: antisense intron1-2: ATTAAGTACAGTAACTCCGT

Generate -/+ 200bp excision

IRF9

gRNA1: sense in exon2: CCGAAAACTCCGGAACTGGG

gRNA2: sense intron2-3: GTGGGATGGTGTATACACAC

Generate -/+ 180bp excision

YY1

gRNA1: sense in exon2: CTGTGCCTAAGTCAAAATGG

gRNA2: sense intron2-3: AGATATTGACCATGAGACAG

Generate -/+ 220bp excision

Supplementary Table 1: gRNAs, exon/intron targets and possible excision size in base pairs (bp) for CRISPR-Cas9
