# Supplementary material for: IRF2 is a master regulator of human keratinocyte stem cell fate
Source: Nat Commun. 2019 Oct 14;10:4676. doi: 10.1038/s41467-019-12559-x (PMC6791852; doi:10.1038/s41467-019-12559-x)
Supplement: Supplementary file 3 — Reporting Summary [file 41467_2019_12559_MOESM3_ESM.pdf]

nature research

Corresponding author(s): Charles Y Lin, Felix Lohmann, Susan Kirkland

Last updated by author(s): Aug 12, 2019

Reporting Summary

Nature Research wishes to improve the reproducibility of the work that we publish. This form provides structure for consistency and transparency in reporting. For further information on Nature Research policies, see Authors & Referees and the Editorial Policy Checklist.

Statistics

For all statistical analyses, confirm that the following items are present in the figure legend, table legend, main text, or Methods section.

n/a	Confirmed	
☐	☒	The exact sample size (*n*) for each experimental group/condition, given as a discrete number and unit of measurement
☐	☒	A statement on whether measurements were taken from distinct samples or whether the same sample was measured repeatedly
☐	☒	The statistical test(s) used AND whether they are one- or two-sided<br>*Only common tests should be described solely by name; describe more complex techniques in the Methods section.*
☐	☒	A description of all covariates tested
☐	☒	A description of any assumptions or corrections, such as tests of normality and adjustment for multiple comparisons
☒	☐	A full description of the statistical parameters including central tendency (e.g. means) or other basic estimates (e.g. regression coefficient) AND variation (e.g. standard deviation) or associated estimates of uncertainty (e.g. confidence intervals)
☐	☒	For null hypothesis testing, the test statistic (e.g. *F*, *t*, *r*) with confidence intervals, effect sizes, degrees of freedom and *P* value noted<br>*Give P values as exact values whenever suitable.*
☒	☐	For Bayesian analysis, information on the choice of priors and Markov chain Monte Carlo settings
☒	☐	For hierarchical and complex designs, identification of the appropriate level for tests and full reporting of outcomes
☒	☐	Estimates of effect sizes (e.g. Cohen's *d*, Pearson's *r*), indicating how they were calculated

Our web collection on statistics for biologists contains articles on many of the points above.

Software and code

Policy information about availability of computer code

Data collection	All data has been produced in-house, except:<br>BRD4-CHIP at Active Motif
Data analysis	>ATAC-seq, ChIP-seq, ChIPmentation. Alignments performed using bowtie2 (version 2.2.8). For ChIP-seq and ChIPmentation, peak calling using MACS 1.4.2. Aligned ATAC-seq reads were processed using RIESLING (https://github.com/GordonLab/riesling-pipeline).<br>>RNA-seq of baseline HSCP-HKs and LSCP-HKs: Fastq files were aligned to the HG19 genome using HiSat2 (version 2.0.4) with default parameters. Transcripts were quantified and FPKM values were generated using cuffquant and cuffnorm from the cufflinks suite (version 2.2.1) (http://cole-trapnell-lab.github.io/cufflinks/) on the HG19 RefSeq transcriptome.<br>>Protein-protein interaction networks: https://string-db.org<br>>IRF2 de novo motif calling: MEME-CHIP (version 4.11.4)<br>>RNA-seq of IRF2 knockdown in HPEKs: As described in S. Schuierer, G. Roma , The exon quantification pipeline (EQP): a comprehensive approach to the quantification of gene, exon and junction expression from RNA-seq data. Nucleic acids research 44, e132 (2016). Differential analysis limma/voom workflow with R version 3.33 (https://cran.r-project.org/doc/FAQ/R-FAQ.html)<br>>CODE AVAILABILITY STATEMENT: All manuscript specific custom analysis scripts and code can be found at: https://github.com/charlesylin/keratinocyte_scripts. The transcriptional core regulatory circuitry analysis code can be found at https://github.com/linlabcode/CRC

For manuscripts utilizing custom algorithms or software that are central to the research but not yet described in published literature, software must be made available to editors/reviewers. We strongly encourage code deposition in a community repository (e.g. GitHub). See the Nature Research guidelines for submitting code & software for further information.

Data

Policy information about availability of data

All manuscripts must include a data availability statement. This statement should provide the following information, where applicable:

- Accession codes, unique identifiers, or web links for publicly available datasets
- A list of figures that have associated raw data
- A description of any restrictions on data availability

Data availability statement (main manuscript)and list of Supplementary Data Files with raw data (Supplementary Information File) have been added to the manuscript

Field-specific reporting

Please select the one below that is the best fit for your research. If you are not sure, read the appropriate sections before making your selection.

☒ Life sciences ☐ Behavioural & social sciences ☐ Ecological, evolutionary & environmental sciences

For a reference copy of the document with all sections, see nature.com/documents/nr-reporting-summary-flat.pdf

Life sciences study design

All studies must disclose on these points even when the disclosure is negative.

Sample size	Sample size calculations were not performed for experiments in this study
Data exclusions	No data points were excluded from the data shown in our mansucript
Replication	>Data was reproduced with neonatal and adult human keratinocytes. Most of the data was reproduced with adult keratinocytes from different donors and at different passages.<br>>All in vitro experiments were performed multiple times with similar outcome<br>> IRF2 finding came from an unbiased CRISPR-Cas9 mini-pool and validated independently
Randomization	Samples were randomly allocated to groups
Blinding	Blinding was not possible in the study. However, experiments involving IRF2 KO were repeating on several occasions following on from the totally unbiased analysis of the CRISPR-Cas9 mini-pool screen

Reporting for specific materials, systems and methods

We require information from authors about some types of materials, experimental systems and methods used in many studies. Here, indicate whether each material, system or method listed is relevant to your study. If you are not sure if a list item applies to your research, read the appropriate section before selecting a response.

Materials & experimental systems

n/a	Involved in the study
☐	☒ Antibodies
☒	☐ Eukaryotic cell lines
☒	☐ Palaeontology
☒	☐ Animals and other organisms
☒	☐ Human research participants
☒	☐ Clinical data

Methods

n/a	Involved in the study
☐	☒ ChIP-seq
☒	☐ Flow cytometry
☒	☐ MRI-based neuroimaging

Antibodies

Antibodies used	Anti-IRF2 (Abcam, cat#ab124744, 1:500 dilution): Anti-IRF9 (Cell Signaling, cat#76684, 1:500 dilution): Anti-YY1 (Cell Signaling, cat#2185, 1:500 dilution). Anti-SNAI2 (Abcam, cat#ab27568, 1:200 dilution). Anti-p16 (CDKN2A, Cell Signaling, cat#ab54210, 1:1000 dilution), Anti-H3K27ac (Active Motif, cat#339685): Anti-HA antibody (Cell Signaling, cat#3724): Anti-cytokeratin 10 (Abcam, cat# ab76318)
Validation	>Anti-IRF2 (Abcam, cat#ab124744): Validated by WB: SW480, HeLa, Jurkat, Caco-2, RAW264.7 and NIH/3T3 cell lysates, and Human fetal lung lysate. IHC-P: Human colon, Human cervical cancer, Mouse colon and human colonic carcinoma tissue. ICC/IF: HeLa cells.<br>>Anti-IRF9 (Cell Signaling, cat#76684): Validated in various publications:<br>A Human Papillomavirus-Independent Cervical Cancer Animal Model Reveals Unconventional Mechanisms of Cervical

Carcinogenesis.
In Cell Reports on 5 March 2019 by He, C., Lv, X., et al..
STAT3 is activated in multicellular spheroids of colon carcinoma cells and mediates expression of IRF9 and interferon stimulated genes.
In Scientific Reports on 24 January 2019 by Edsbäcker, E., Serviss, J. T., et al..
Bclaf1 critically regulates the type I interferon response and is degraded by alphaherpesvirus US3.
In PLoS Pathogens on 1 January 2019 by Qin, C., Zhang, R., et al..
ISGF3 with reduced phosphorylation is associated with constitutive expression of interferon-induced genes in aging cells.
In Npj Aging and Mechanisms of Disease on 21 November 2018 by Yamagami, M., Otsuka, M., et al..
Multiple tumor suppressors regulate a HIF-dependent negative feedback loop via ISGF3 in human clear cell renal cancer.
In eLife on 25 October 2018 by Liao, L., Liu, Z. Z., et al..
DNA-PK inhibition synergizes with oncolytic virus M1 by inhibiting antiviral response and potentiating DNA damage.
In Nature Communications on 18 October 2018 by Xiao, X., Liang, J., et al..
Interferon-Stimulated Genes Are Transcriptionally Repressed by PR in Breast Cancer.
In Molecular Cancer Research on 1 October 2017 by Walter, K. R., Goodman, M. L., et al..
Methyltransferase SETD2-Mediated Methylation of STAT1 Is Critical for Interferon Antiviral Activity.
In Cell on 27 July 2017 by Chen, K., Liu, J., et al..
>Anti-YY1 (Cell Signaling, cat#2185) Validated by WB in 5 papers:
In Oncotarget on 13 June 2017 by Ye, X., Zhang, Y., et al..
WB (1:1000)
miR-34 and p53: New Insights into a Complex Functional Relationship.
In PLoS ONE on 16 July 2015 by Navarro, F. & Lieberman, J..
Transcriptional repression of Bim by a novel YY1-RelA complex is essential for the survival and growth of Multiple Myeloma.
In PLoS ONE on 23 July 2013 by Potluri, V., Noothi, S. K., et al..
Transcription factor Foxp3 and its protein partners form a complex regulatory network.
In Nature Immunology on 1 October 2012 by Rudra, D., DeRoos, P., et al..
A role for YY1 in repression of dominant negative LEF-1 expression in colon cancer.
In Nucleic Acids Research on 1 October 2010 by Yokoyama, N. N., Pate, K. T., et al..
>Anti-SNAI2 (Abcam, cat#ab27568)
In house validated gave a positive signal in the following tissue lysates: Mouse heart, Rat heart as well as MCF7 cell lysate overexpressing SLUG protein. Validated in over 70 publications (https://www.abcam.com/slug-antibody-ab27568-references.html#active-tab)
>Anti-p16 (CDKN2A, Cell Signaling, cat#ab54210): Validated in house using Human brain tumor, ovarian carcinoma, cervix, skin and brain tissue. Rat liver tissue. Flow Cyt: HeLa cells. Validated in over 44 publications (https://www.abcam.com/cdkn2ap16ink4a-antibody-2d9a12-ab54210-references.html#active-tab)
>Anti-H3K27ac (Active Motif, cat#339685)
Validated by ChIP in 8 publications:
Coming soon: Experimental images - join our mailing list for updates
The long non-coding RNA Kcnq1ot1 controls maternal p57 expression in muscle cells by promoting H3K27me3 accumulation to an intragenic MyoD-binding region.
In Epigenetics & Chromatin on 16 January 2019 by Andresini, O., Rossi, M. N., et al..
EZH2 Inhibition by Tazemetostat Results in Altered Dependency on B-cell Activation Signaling in DLBCL.
In Molecular Cancer Therapeutics on 1 November 2017 by Brach, D., Johnston-Blackwell, D., et al..
EZH2 enables germinal centre formation through epigenetic silencing of CDKN1A and an Rb-E2F1 feedback loop.
In Nature Communications on 12 October 2017 by Béguelin, W., Rivas, M. A., et al..
Retinoic acid controls body axis extension by directly repressing Fgf8 transcription.
In Development (Cambridge, England) on 1 August 2014 by Kumar, S. & Duester, G..
Ezh2 regulates transcriptional and posttranslational expression of T-bet and promotes Th1 cell responses mediating aplastic anemia in mice.
In The Journal of Immunology on 1 June 2014 by Tong, Q., He, S., et al..
Selective inhibition of EZH2 by EPZ-6438 leads to potent antitumor activity in EZH2-mutant non-Hodgkin lymphoma.
In Molecular Cancer Therapeutics on 1 April 2014 by Knutson, S. K., Kawano, S., et al..
Myelodysplastic syndromes are induced by histone methylation–altering ASXL1 mutations.
In The Journal of Clinical Investigation on 1 November 2013 by Inoue, D., Kitaura, J., et al..
A transcriptional repressor co-regulatory network governing androgen response in prostate cancers.
In The EMBO Journal on 13 June 2012 by Chng, K. R., Chang, C. W., et al..

> Anti-HA antibody (Cell Signaling, cat#3724)
Validated in 5 citations:
BRD9 defines a SWI/SNF sub-complex and constitutes a specific vulnerability in malignant rhabdoid tumors.
In Nature Communications on 23 April 2019 by Wang, X., Wang, S., et al..
ChIP (1:3000) and IP (1:3000)
H3K27M induces defective chromatin spread of PRC2-mediated repressive H3K27me2/me3 and is essential for glioma tumorigenesis.
In Nature Communications on 19 March 2019 by Harutyunyan, A. S., Krug, B., et al..
None Available
Targeted degradation of BRD9 reverses oncogenic gene expression in synovial sarcoma.
In eLife on 15 November 2018 by Brien, G. L., Remillard, D., et al..
Targeting IRF3 as a YAP agonist therapy against gastric cancer.
In The Journal of Experimental Medicine on 5 February 2018 by Jiao, S., Guan, J., et al..
AKR1C1 Activates STAT3 to Promote the Metastasis of Non-Small Cell Lung Cancer.
In Theranostics on 19 January 2018 by Zhu, H., Chang, L. L., et al..
>Anti-cytokeratin 10 (Abcam, cat# ab76318)
In house validated for IHC-P using Human skin and tonsil tissues and mouse skin tissue. ICC/IF: HACAT cells
Validated in 23 publications (https://www.abcam.com/cytokeratin-10-antibody-ep1607ihcy-cytoskeleton-marker-ab76318-

references.html#active-tab
>Anti-BRD4 Bethyl (A301-985A100-6):
This antibody has been validated by ChIP in 12 publications (https://www.citeab.com/antibodies/1231153-a301-985a100-brd4-antibody?utm_campaign=Widget+All+Citations&utm_medium=Widget&utm_source=Bethyl+Laboratories&utm_term=Bethyl+Laboratories)

ChIP-seq

Data deposition

☒ Confirm that both raw and final processed data have been deposited in a public database such as GEO.

☐ Confirm that you have deposited or provided access to graph files (e.g. BED files) for the called peaks.

Data access links<br>*May remain private before publication.*	High throughput sequencing data that support the findings of this study have been deposited in NCBI GEO (www.ncbi.nlm.nih.gov/geo) under the overall accession code: GSE135680. ATAC-seq in HSCP-HKs and LSCP-HKs can be found under: GSE135675. H3K27ac and BRD4 ChIP-seq in HSCP-HKs and LSCP-HKs can be found under: GSE135676. IRF2-HA ChIPmentation can be found under: GSE135677. RNA-seq of baseline HSCP-HKs and LSCP-HKs can be found under: GSE135679. RNA-seq expression level count data for IRF2-KD experiments are available in Supplementary Data Files 5 and 6. Peak files are not associated with GEO, uploaded to https://github.com/charlesylin/keratinocyte_scripts/
Files in database submission	For ChIP-seq, ATAC-seq, ChIPmentation – raw reads (fastqs) and pileups (.wig) are provided. For RNA-seq in baseline HSCP-HKs and LSCP-HKs – raw reads (fastqs) and count files (fpkm) are provided)
Genome browser session<br>(e.g. UCSC)	N/A

Methodology

Replicates	H3K27ac ChIP performed in triplicate<br>BRD4 ChIP performed in duplicate<br>IRF2-HA ChIPmentation performed in duplicate
Sequencing depth	Supplementary Data File 7 contains sequencing depth information for all samples
Antibodies	BRD4 ChIP-Active Motif performed the BRD4 ChIP with a BRD4 antibody from Bethyl Labs rabbit anti-BRD4 pAb cat # A301-985A100-6 (= lot # 6).<br>H3K27ac ChIP performed with anti-H3K27ac (Active Motif, cat#339685)
Peak calling parameters	MACS 1.4.2 with a p-value 1e-9 cutoff
Data quality	FASTQC (https://www.bioinformatics.babraham.ac.uk/projects/fastqc/) for raw read quality. Custom analysis for processed data quality
Software	All manuscript specific custom analysis scripts and code can be found at: https://github.com/charlesylin/keratinocyte_scripts. The transcriptional core regulatory circuitry analysis code can be found at https://github.com/linlabcode/CRC.
