# Supplementary material for: IRF2 is a master regulator of human keratinocyte stem cell fate
Source: Nat Commun. 2019 Oct 14;10:4676. doi: 10.1038/s41467-019-12559-x (PMC6791852; doi:10.1038/s41467-019-12559-x)
Supplement: Supplementary file 4 — Description of Additional Supplementary Files [file 41467_2019_12559_MOESM4_ESM.pdf]

Description of Additional Supplementary Files

Supplementary Data 1: RNA-seq data (HSCP-HK vs LSCP-HK)

Supplementary Data 2: List of Gene Set Analysis (GSA) genes enrichment for LSCP-HK vs HSCP-HK

Supplementary Data 3: Effect of gene editing on cell proliferation at Days 12, 19, 38 and 43 (Q1/3, RSA)

Supplementary Data 4: Gain in IRF2 binding at regulatory regions of genes (log2FC < 0.5) in LSCP-HK vs HSCP-HKs

Supplementary Data 5: Counts from RNA-seq LSCP-HK vs HSCP-HKs

Supplementary Data 6: Counts from RNA-seq IRF2 KO vs control

Supplementary Data 7: HG19 human keratinocyte sequencing reads table
